# Patient‐reported outcomes for monitoring substance use treatment: A systematic review of single‐item measures

**DOI:** 10.1111/add.70424

**Published:** 2026-04-22

**Authors:** Thomas J. Reese, Hilary A. Tindle, Justin Bachmann, Adam Wright, Jessica S. Ancker, Carolyn M. Audet, Mauli V. Shah, Bryan D. Steitz, Michael H. Levin, Kristopher A. Kast, David Marcovitz, Amanda von Horn, A. Taylor Kelley, John F. P. Bridges

**Affiliations:** ^1^ Department of Biomedical Informatics Vanderbilt University Medical Center Nashville TN USA; ^2^ Department of Medicine, Division of Internal Medicine and Public Health Vanderbilt University Medical Center Nashville TN USA; ^3^ Department of Veterans Affairs Tennessee Valley Health System Geriatric Research and Education Center (GRECC) for Healthy Longevity Nashville TN USA; ^4^ Department of Physical Medicine and Rehabilitation Vanderbilt University Medical Center Nashville TN USA; ^5^ Department of Health Policy Vanderbilt University Medical Center Nashville TN USA; ^6^ Department of Psychiatry and Behavioral Science Vanderbilt University Medical Center Nashville TN USA; ^7^ Division of General Internal Medicine University of Utah Salt Lake City UT USA; ^8^ Department of Biomedical Informatics Ohio State University Wexner Medical Center Columbus OH USA

**Keywords:** addiction, COSMIN, measurement‐based care, patient‐reported outcomes, psychometric properties, reliability, single‐item measures, substance use disorder, systematic review, validity

## Abstract

**Background and aims:**

Measurement‐based care (MBC) is a structured approach using standardized, repeated assessments to monitor treatment progress and guide clinical decision‐making. MBC improves outcomes for substance use treatment but can be time consuming due in part to lengthy assessment tools. Single‐item, patient‐reported outcome measures (PROMs) offer a more acceptable alternative for routine monitoring, yet their psychometric properties have not been systematically evaluated. We sought to identify constructs assessed by single‐item PROMs in substance use treatment and critically appraise their validity, reliability and overall quality using standardized criteria.

**Methods:**

We conducted a systematic review following COSMIN and PRISMA guidelines. MEDLINE, Embase and PsycINFO were searched from January 2005 to August 2025 for studies evaluating single‐item PROMs in adults with substance use. We assessed psychometric properties, including content validity, test–retest reliability, construct validity, responsiveness and predictive validity using COSMIN criteria. Quality of evidence was assessed using a modified GRADE approach.

**Results:**

Of 4722 records screened, 35 studies met inclusion criteria, evaluating 68 single‐item PROMs across 9 clinical constructs for more than 50 000 participants. Fifteen studies achieved an overall rating of sufficient measure properties and moderate‐or‐above level of evidence rating across domains. Test–retest reliability ranged approximately from Intraclass Correlation Coefficient = 0.60–0.85; construct validity correlations approximately ranged r = 0.11–0.98. Predictive validity was strong for several measures, with odds ratios up to 7.3 for treatment readiness. Measures assessing craving, treatment readiness and self‐efficacy demonstrated the most robust evidence and, in some cases, outperformed multi‐item scales. However, over half of measures lacked empirically validated thresholds and responsiveness to change analyses, limiting clinical interpretability and treatment monitoring.

**Conclusions:**

Single‐item patient‐reported outcome measures (PROMs) are pragmatic tools for implementing measurement‐based care in substance use treatment, offering strong implementation feasibility and, in some cases, predictive performance comparable to longer instruments. PROMs lacking validated thresholds or responsiveness may be best used as complementary tools, whereas those with strong evidence and thresholds can support primary monitoring.

## INTRODUCTION

Measurement‐based care (MBC) is a systematic approach to healthcare that uses standardized, repeated assessments to monitor treatment progress and guide clinical decision‐making in real time [1, 2]. MBC has demonstrated benefits in mental health and substance use treatment. A literature review by Fortney and colleagues found that studies reported up to a nearly 75% improvement in remission rates among patients receiving MBC for behavioral health conditions, compared with those who received usual care [3]. The authors noted that most randomized controlled trials featuring frequent and timely feedback of patient‐reported symptoms to providers during medication management and psychotherapy encounters significantly improved outcomes. In substance use treatment specifically, feedback using Outcome Questionnaire‐45 (OQ‐45) for patients considered ‘off‐track’ reduced drug and alcohol use [4]. However, the adoption of MBC remains disappointingly limited, in part because of the burden of lengthy assessment batteries, often including irrelevant items, and the difficulty in translating results into treatment decisions [5, 6, 7]. This paradox highlights the challenge of translating evidence‐based practices into feasible and useful clinical applications. Single‐item patient‐reported outcome measures (PROMs) are inherently more feasible and acceptable to patients and clinicians, potentially achieving the dual goals of monitoring and clinical utility; despite this, the evidence remains fragmented and lacks systematic evaluation using standardized psychometric criteria [8, 9, 10].

Single‐item PROMs can achieve notable predictive capabilities, with performance comparable with multi‐item scales. For example, single‐item self‐efficacy measures have outperformed established 20‐item scales in predicting substance use relapse and have comparable discriminative performance to that of comprehensive instruments for mortality prediction [11, 12, 13]. However, the psychometric evaluation of many single‐item PROMs is either lacking critical aspects of validity or absent entirely. No comprehensive systematic review has applied the COSMIN (COnsensus‐based Standards for the selection of health Measurement INstruments) guidelines to evaluate single‐item PROMs in substance use treatment, creating uncertainty about when and how to appropriately implement these single‐item PROMs [14, 15, 16, 17]. In this review, we use the term ‘substance use treatment’ broadly, to encompass both formal substance use disorder treatment and clinical care for unhealthy substance use that may not meet diagnostic thresholds, reflecting the range of populations included in the underlying studies.

Single‐item PROMs are uniquely positioned to address challenges of substance use treatment, including non‐linear recovery trajectories that require frequent monitoring, high‐volume treatment settings that demand efficient assessment approaches, and patient‐centered recovery trajectories that align well with personalized interpretation [18, 19]. Their high feasibility profile, including administration times of under 2 minutes, minimal rates of missing data, freedom from licensing fees, intuitive interpretation and minimal burden on clinical workflows, makes them particularly well suited for the routine monitoring that is essential to effective MBC implementation. Not all constructs (e.g. recovery and relapse risk), however, have been amenable to single‐item PROMs. Multi‐dimensional or abstract constructs, such as depression, benefit from comprehensive assessment across dimensions (e.g. emotional symptoms and cognitive symptoms) for diagnosis and longitudinal treatment [20]. Furthermore, single‐item PROMs may not provide requisite validity and reliability for constructs serving as primary study end‐points [8, 21]. For example, assessing ‘overall health’ with a single question, such as ‘How healthy do you feel?’, may fail to capture important aspects such as physical functioning, mental wellbeing and social health. While concrete, uni‐dimensional constructs are ideal for single‐item PROMs, the application of these measures to conventionally multi‐dimensional constructs such as substance use disorder can be problematic [22, 23]. Without psychometric studies demonstrating the effectiveness for certain constructs, it is unclear which constructs are amenable [24, 25].

This study addresses a key barrier to implementing evidence‐based measurement practices in addiction treatment: the limited evaluation of single‐item PROMs for substance use monitoring [26]. Guided by COSMIN standards, we synthesized evidence across multiple constructs and populations to inform MBC implementation. This review intentionally examines a broad spectrum of constructs relevant to substance use and recovery, recognizing that the availability and quality of single‐item PROMs vary substantially across substances and constructs. By providing evidence‐based recommendations for selecting appropriate measures, identifying research gaps and supporting precision medicine approaches, this review aims to improve patient outcomes while maintaining feasibility for routine clinical practice [27]. Our objectives for this study were to: (i) identify the constructs assessed by single‐item PROMs used in the context of substance use treatment; and (ii) critically appraise, compare and synthesize evidence on the reliability, validity, responsiveness and overall quality of these single‐item PROMs. Unlike prior reviews that emphasize multi‐item instruments or access barriers, this review systematically evaluates single‐item PROMs using COSMIN and a modified GRADE (Grading of Recommendations Assessment, Development, and Evaluation) approach, with specific attention given to routine monitoring use [16, 28, 29, 30].

## METHODS

### Study design and registration

In this systematic review, we aimed to identify constructs assessed by single‐item PROMs for substance use, and to evaluate their measurement properties in accordance with the COSMIN guidelines for systematic reviews of PROMs and align the findings with the PRISMA (Preferred Reporting Items for Systematic Reviews and Meta‐Analyses) statement [14, 15, 31, 32]. The review protocol was prospectively registered with PROSPERO (CRD420251141071).

### Search strategy

A comprehensive search strategy was developed and implemented across multiple electronic databases from January 2005 through August 2025. We searched MEDLINE (via PubMed), Embase and PsycINFO using a combination of controlled vocabulary terms and keywords related to: (i) substance use; (ii) single‐item PROMs or brief assessment tools; (iii) psychometric properties; and (iv) measurement‐based care or treatment monitoring. The search strategy combined substance‐specific terms (alcohol, cannabis, cocaine, opioids, stimulants, tobacco) with measurement methodology terms following established COSMIN search filters. Key search terms included: ‘substance use disorder’, ‘addiction’, ‘single‐item’, ‘brief measure’, ‘patient‐reported outcome’, ‘psychometric’, ‘reliability’, ‘validity’, ‘responsiveness’ and ‘measurement‐based care’. The complete search strategy for PubMed is provided in Table S1.

### Eligibility criteria and study selection

We included studies if they evaluated single‐item PROMs designed for monitoring adults (aged ≥18 years) across all substance classes (alcohol, cannabis, cocaine, opioids, amphetamine‐type stimulants, tobacco and other substances). Eligible studies needed to report at least one psychometric property, including content validity, test–re‐test reliability, construct validity, responsiveness, predictive validity or interpretability using multi‐item measures, clinical assessments or objective outcomes as reference standards for psychometric evaluation. We considered multi‐item measures if the constructs were independently evaluated. Quantitative studies reporting original empirical data on measurement properties, encompassing cross‐sectional, longitudinal, randomized controlled trials, and observational studies were considered for inclusion. For studies in languages other than English, we translated the title and abstract and sought full translation if necessary. We excluded studies if they focused solely on screening or diagnostic assessment rather than treatment monitoring, assessed only behavioral or process addictions, evaluated multi‐item scales or composite measures, included only adolescent populations (<18 years), were non‐peer‐reviewed publications, reviews, commentaries or case reports, or did not report sufficient data for psychometric evaluation. Two reviewers assessed titles and abstracts on Covidence using predefined criteria, with disagreements resolved through discussion, reference to the original source documents or consultation with a third reviewer. Full‐text articles were subsequently reviewed, and data were extracted and synthesized using a similar process with two reviewers.

### Data extraction

We extracted data from included studies using a standardized form developed based on COSMIN recommendations. For each included study, we extracted study characteristics (first author; publication year; PubMed identifier or digital object identifier, DOI; study design; setting; sample size; population demographics), instrument characteristics (measure name; substance type; concept measured; measure wording; response format; administration method; completion time), psychometric properties (Box 1) and feasibility indicators (completion time; missing data rates; administration mode; cost; licensing requirements).

Box 1 **Assessed COSMIN (COnsensus‐based Standards for the selection of health Measurement INstruments) psychometric domains and items.**

**Table:** 

Domains	Items
Content validity	Method used to assess content validity, involvement of target population, expert involvement, relevance, comprehensiveness, comprehensibility
Test–re‐test reliability	Sample size, time interval, measure, anchor used to define stability, methodological quality
Construct validity	Comparison measure, hypothesized relationship, observed correlation, type of validity assessed, hypothesis confirmed, methodological quality
Known‐groups validity	Groups compared, hypothesized difference, statistical test used, difference observed, effect size, methodological quality
Responsiveness	Change scores compared, correlation of change, effect size, hypotheses confirmed, methodological quality
Predictive validity	Outcome predicted, comparator, measure association, time frame, methodological quality
Interpretability	Score distribution, floor/ceiling effects, meaningful change threshold, method used

### Quality assessment

The methodological quality of each study and the quality of the measurement properties were evaluated using the COSMIN Risk of Bias checklist. For each single‐item PROM, we first assessed the quality for each psychometric property (Box 1) using updated criteria for good measurement properties (sufficient, +; insufficient, –; indeterminate, ?). Subsequently, we pooled results across measurement domains for each study using predefined decision rules: (i) ‘sufficient’ (+) if >75% of study results were rated sufficient; (ii) ‘insufficient’ (–) if >75% of study results were rated insufficient; (iii) ‘inconsistent’ (±) if study results were split without adequate explanation for heterogeneity; and (iv) ‘indeterminate’ (?) if all individual study results were indeterminate. Overall quality ratings were assigned using the modified GRADE approach adapted for measurement instruments, considering risk of bias, inconsistency, imprecision and indirectness to assign overall evidence quality ratings of high, moderate, low or very low [33].

### Data synthesis

Given the heterogeneity in study designs, populations and measurement approaches, we conducted a narrative synthesis following established guidelines [34]. Psychometric properties were synthesized across seven domains (Box 1). For each single‐item PROM, reviewers extracted and evaluated evidence using the COSMIN checklist and applied standardized decision rules to assign ratings of ‘sufficient’, ‘insufficient’, ‘indeterminate’ or ‘inconsistent’ per property.

We developed a comprehensive evidence matrix, which included item‐level data for each psychometric domain. This matrix captured methodological quality, statistical outcomes and COSMIN ratings for each study and measure. Summary tables in the manuscript were derived directly from this matrix, allowing for cross‐domain comparison and the identification of high‐performing instruments. We did not perform a meta‐analysis owing to variability in reference standards and outcome measures. We synthesized interpretability data, including score distributions, floor/ceiling effects and clinically meaningful change thresholds. Methods to determine thresholds (e.g. anchor‐ and distribution‐based methods) were noted where available. Measures with empirically derived clinical cut‐points were highlighted to support implementation in MBC.

We organized the results by psychometric property and synthesized the data within clinical constructs (e.g. craving, self‐efficacy, quality of life, motivation) and substance (e.g. alcohol, opioids). Evidence synthesis utilized both the quality of individual studies and the consistency of findings across studies. We identified patterns in psychometric performance and highlighted gaps where evidence was limited or indeterminate. Summary tables were constructed to facilitate comparison across measures and domains, with COSMIN and modified GRADE ratings providing standardized quality indicators for each psychometric property.

## RESULTS

Of 4722 unique studies identified, 35 studies met the inclusion criteria, evaluating 68 single‐item PROMs across nine clinical constructs in adults who use substances (Figure S1) [11, 25, 35, 36, 37, 38, 39, 40, 41, 42, 43, 44, 45, 46, 47, 48, 49, 50, 51, 52, 53, 54, 55, 56, 57, 58, 59, 60, 61, 62, 63, 64, 65, 66, 67]. These constructs included assessments of craving/urge intensity [35, 42, 43, 48, 49], treatment readiness [36, 40, 41, 50, 56, 62, 63, 64], self‐efficacy/confidence [11, 44, 55, 60, 61, 65], substance use patterns [39, 53, 67], quality of life [25, 38], risk perception [45, 52, 59], withdrawal monitoring [47, 54], dependence/addiction [46, 51, 57, 58] and recovery progress [37, 66]. Sample sizes ranged from five to >32 000, spanning alcohol [39, 40, 41, 44, 49, 50, 55, 56, 60, 64], cannabis [53, 62], cocaine [46], methamphetamine [66], opioids [35, 37, 47, 59], tobacco/nicotine [42, 51, 52, 54, 57, 58, 61, 63, 67] and general/multiple substances [11, 25, 36, 38, 43, 45, 48, 65]. All measures took less than 2 minutes to administer, demonstrated minimal missing data (<10%) and were free of licensing fees.

**FIGURE 1:**
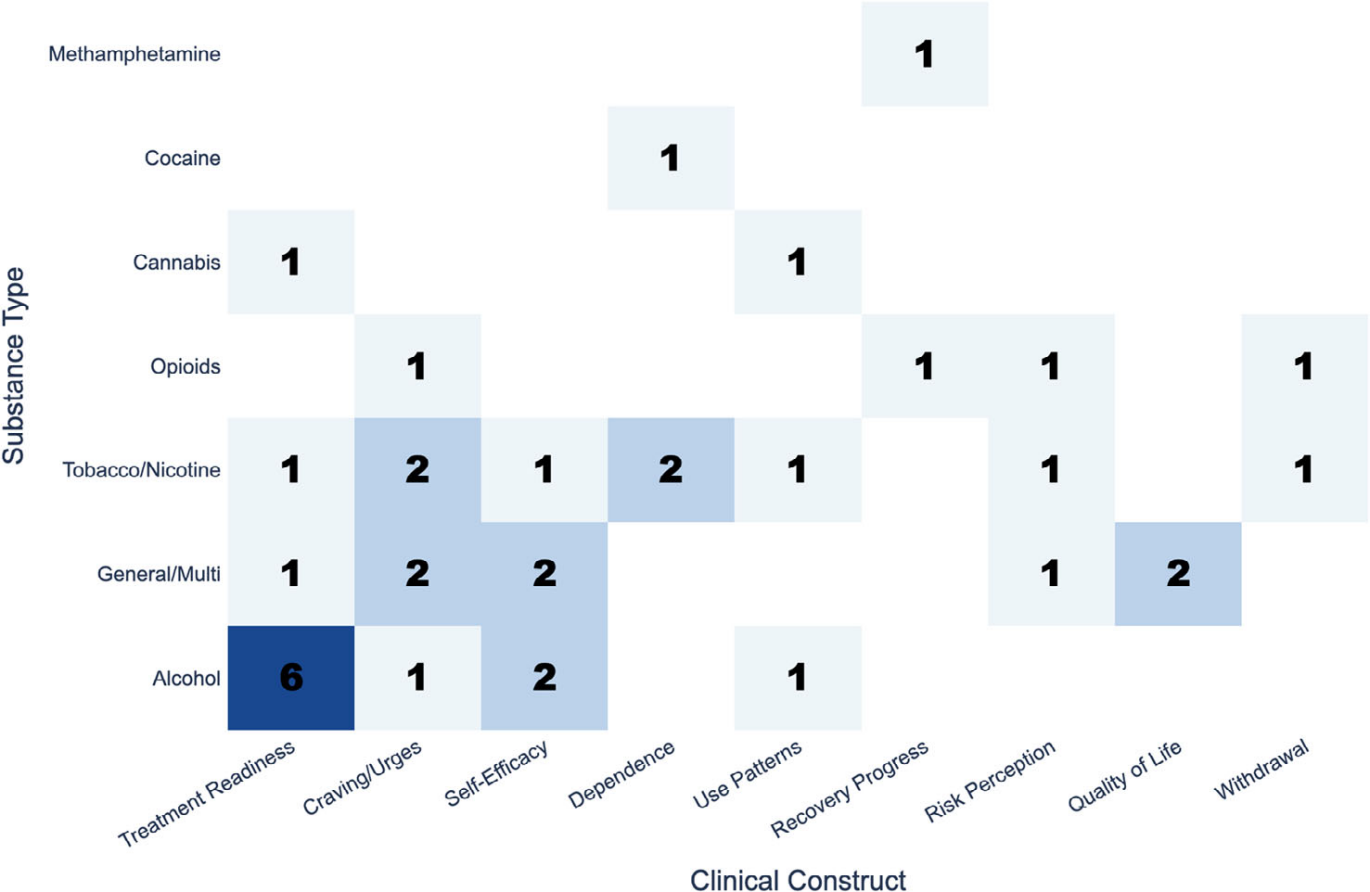
Coverage of single‐item patient‐reported outcome measure studies across substance type and clinical construct. Darker boxes indicate a higher number of studies.

**FIGURE 2:**
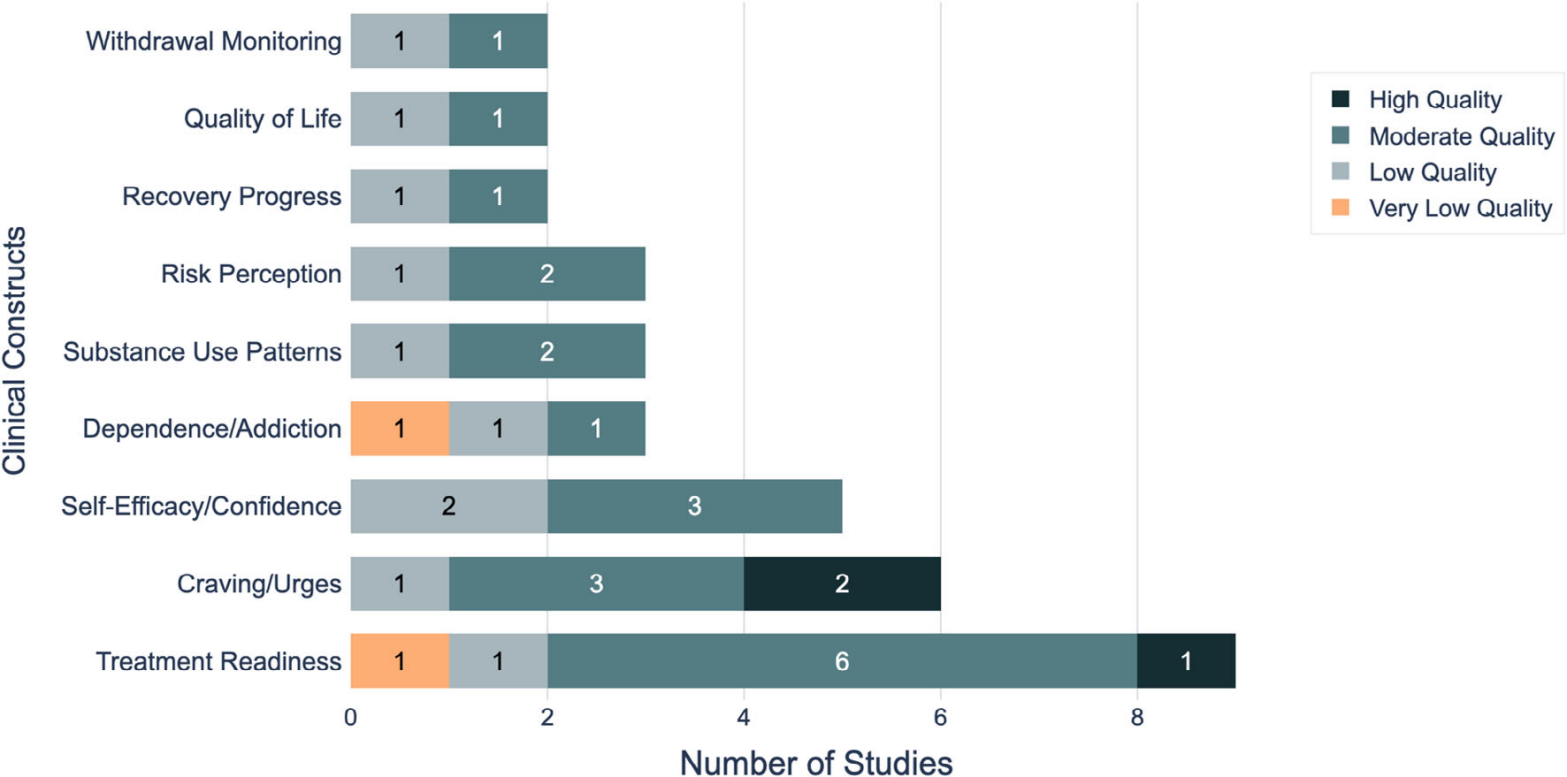
Evidence quality distribution by clinical construct. Darker boxes indicate higher quality and the number in the box indicates the number of studies.

Substances covered by single‐item PROMs include alcohol, cannabis, cocaine, methamphetamine, opioids, tobacco/nicotine and general/multiple substances (Figure 1; Table 1). Among the 35 included studies, the quality of evidence was variable, with alcohol and tobacco/nicotine having the most studies and the strongest overall evidence base, while treatment readiness and craving assessment represented the most extensively studied constructs (Figure 2). Tobacco/nicotine covered the most clinical constructs, including dependence/addiction [51, 57], risk perception [52], substance use patterns [67], withdrawal monitoring [54], craving/urges [42, 58], treatment readiness [63] and self‐efficacy/confidence [61]. Cravings/urges were included in most substance categories, with the strongest evidence for general/multiple substances [48] and opioids [35]. Most treatment readiness analyses were for alcohol‐related measures, with measures from Bertholet *et al*. showing the highest quality of evidence [64]. Quality of life was only covered in the general/multiple substances category, with the measure by Laudet et al. showing predictive validity in a population using multiple illicit drugs [25]. Responsiveness to change, a key property for MBC, was sufficiently demonstrated in six single‐item PROMs, with generally adequate effect sizes and methodological quality supporting their utility in monitoring progress [35, 37, 45, 47, 48, 50]. These measures spanned clinical constructs including craving, recovery progress, withdrawal symptoms, risk perception and treatment readiness, and were validated across varied populations and settings. However, responsiveness data often remain limited for other measures.

**TABLE 1:** Substance‐specific measures and recommendations.

Author (year)	Clinical construct	Patient population	Instrument	Key outcomes predicted	Evidence quality (overall)	COSMIN rating (overall)	COSMIN (summary)
**Alcohol**							
Bertholet (2009)	Treatment readiness	Medical in‐patients with unhealthy alcohol use (*n* = 267, mean age 45.0 ± 10.5 years)	VAS (1–10): readiness; importance; confidence	Average drinks per day at 3 months	Moderate	+	Strong evidence for predictive validity of taking action; VAS useful at high threshold
Demmel (2006)	Self‐efficacy/confidence	Alcohol‐dependent European in‐patients (*n* = 142)	Confidence (0–10)	Abstinence/relapse at 12 weeks following discharge	Moderate	+	Strong evidence for construct validity and interpretability; predictive validity limited
Hogue (2010)	Treatment readiness	Adult welfare recipients with SUD (*n* = 394)	Contemplation ladder (1–7)	Days abstinent, SUD treatment	Moderate	±	Strong for predictive/convergent; widely validated in addiction
Ludwig (2013)	Self‐efficacy/confidence	Adults in residential AUD treatment (*n* = 415)	Self‐efficacy (1–10)	Abstinence at 1 year, time to drink	Moderate	±	Strong for clinical prediction
Maisto (2011)	Treatment readiness	Adolescents in outpatient SUD treatment (*n* = 161)	Readiness Ruler (1–10)	Percentage of days abstinent, drinks per drinking day	Moderate	±	Strong for predictive use, practical in adolescent settings
Dollinger (2009)	Substance use patterns	College students (*n* = 164, USA, aged 18–54 years)	Alcohol frequency/quantity	College GPA, study log, behavior	Moderate	±	Strong for reliability and construct validity; limited for responsiveness and predictive validity
Williams (2007)	Treatment readiness	Primary care patients with unhealthy alcohol use (*n* = 228)	Items: readiness (1–10); importance (1–10); confidence (1–10); intention to cut down (1–5); intention to abstain (1–5)	Heavy episodic drinking; abstinence/reduced drinking; consequences (Short Inventory of Problems, v2)	Moderate	±	Moderate: valid for brief assessment and prediction; confidence item most promising
Heather (2008)	Treatment readiness	Adults with hazardous/harmful alcohol use in UK primary care (*n* = 72)	Readiness Ruler (0–4)	Not assessed in this paper	Moderate	±	Strong initial support; well‐developed for practical brief intervention settings
Rosenberg (2007)	Craving/urges	University students (*n* = 122) with ≥2 binge episodes in last 30 days	VAS (0–100): need; desire; craving; urge; compulsion	Drinking frequency, drinks/drinking day, binge episodes	Low	±	Moderate: useful for brief craving assessment in non‐clinical populations; limited psychometric depth
Piontek (2017)	Treatment readiness	329 adult in‐patients (Germany): alcohol (*n* = 177) and drug (*n* = 152) dependent	VAS readiness (0–10)	No direct outcome prediction (e.g. abstinence, retention)	Low	±	Moderate: useful for tracking individual treatment readiness over time; limited psychometric depth
**Cannabis**							
Caviness (2013)	Treatment readiness	Young adult women marijuana users (*n* = 332, aged 18–24 years)	Change ladder (1–10)	Marijuana use reduction/cessation	Moderate	+	Strong evidence for construct and predictive validity; gaps in reliability and responsiveness
Norberg (2012)	Substance Use Patterns	Adult cannabis users (Australia, *n* = 98)	Cannabis use quantity and frequency	Cannabis Problems Questionnaire and Severity of Dependence Scale	Moderate	+	Strong evidence for reliability (ICC up to 0.97), validity (convergent, discriminant, incremental) and interpretability; gaps only in content validity and responsiveness
**Cocaine**							
Yoon (2021)	Dependence/addiction	Adults (*n* = 22) with moderate–severe DSM‐5 cocaine use disorder, urban USA	Brief assessment of cocaine demand	Not assessed	Very low	±	Moderate: strong construct validity; limited evidence for other psychometric domains
**General/multiple SUD**							
Shram (2020)	Craving/urges	Healthy adult recreational drug users, *n* = 570 (across 19 studies, 7 opioid)	VAS (0–100): drug liking; bad effects; good effects	Post‐market abuse rates (Food and Drug Administration Adverse Event Reporting System)	High	+	High: strong evidence across most psychometric domains; recommended as a core measure
Laudet (2009)	Quality of life	Adults with chronic/severe dependence to crack, heroin or multiple illicit drugs; urban USA (*n* = 289)	Quality of life satisfaction (1–10)	Sustained remission from illicit drug use	Moderate	+	Strong for predictive validity; limited for reliability and responsiveness
Bertholet (2012)	Self‐efficacy/confidence	Swiss young men (*n* = 461, aged approx. 20 years) with unhealthy alcohol use and/or smoking	VAS (readiness, importance, confidence)	Drinks per day at 3 months	Moderate	+	Strong evidence for predictive validity of confidence; readiness less predictive
Heinz (2006)	Craving/urges	Outpatients with cocaine or heroin dependence (*n* = 173)	VAS craving (0–100)	Drug use in treatment	Moderate	+	Moderate: valid for brief craving assessment; multi‐dimensional tools may offer richer insights
Hoeppner (2011)	Self‐efficacy/confidence	Young adults (aged 18–24 years) in residential SUD treatment (*n* = 303)	Single‐item self‐efficacy (0–10)	Relapse to any substance use	Low	±	Strong for construct validity; limited for reliability and responsiveness
Blanchard (2023)	Risk perception	Adults with SUD, Veteran Affairs (*n* = 32 002)	Brief Addiction Monitor‐Revised (5 items)	SUD treatment retention; 12‐month mortality; as above	Low	±	Moderate: strong responsiveness and feasibility; limited evidence for other psychometric domains
Muller (2016)	Quality of life	Adults in SUD treatment in Norway (*n* = 107)	Quality of Life 1	Not assessed	Low	±	Valid as a brief global QoL item; limited psychometric data
Smith (2017)	Treatment readiness	Emerging adults (aged 18–25 years) with SUD (*n* = 1951)	Readiness to remain abstinent/stop using (0–100%)	Abstinence and early remission status	Very low	±	Limited evidence, primarily for predictive validity; other properties largely unexamined by this study
**Methamphetamine**							
Vo (2023)	Recovery progress	Adults with moderate to severe methamphetamine use disorder (*n* = 403) in ADAPT‐2 trial	Treatment Effectiveness Assessment	Not assessed	Low	±	Acceptable for validity; predictive and responsiveness evidence lacking
**Opioids**							
Boyett (2021)	Craving/urges	Adults with moderate/severe OUD (*n* = 487)	Opioid craving VAS (0–100)	Opioid use during treatment	High	+	Strong evidence for use in OUD populations
Eaton (2012)	Risk perception	Adults in opioid and heroin lab studies (variable *n*, e.g. 5–60)	Drug high VAS (0–100)	Clinical outcome: drug self‐administration, retention	Moderate	+	Strong evidence for clinical interpretability and predictive validity of VAS drug high scores; CID established at 8–10 mm; strong anchor‐based validation compensates for other gaps
Ling (2019)	Recovery progress	Adults with moderate/severe OUD in BUP‐XR clinical trial (*n* = 410)	Treatment Effectiveness Assessment	≥80% abstinent during treatment	Moderate	+	Good overall across domains (use, health, lifestyle, community)
Tompkins (2009)	Withdrawal monitoring	Adults with opioid dependence (*n* = 46)	VAS: ‘bad effects’ and ‘sick’ (0–100)	Not assessed	Low	±	Moderate: valid for acute withdrawal detection; limited evidence for test–re‐test and long‐term or predictive use
**Tobacco/nicotine**							
Bienkowski (2015)	Treatment readiness	Post‐stroke smokers (*n* = 86, White, active smokers with first‐ever ischemic stroke)	VAS readiness (0–10)	Continued smoking vs cessation	High	+	Strong evidence for predictive validity and reliability; gaps in responsiveness
Fidler (2010)	Craving/urges	National sample of English smokers (*n* > 15 000)	Strength of urges to smoke (0–5)	Smoking relapse after quit attempt	Moderate	+	Good: strong predictive utility for monitoring; outperformed Fagerström Test for Nicotine Dependence/Heaviness of Smoking Index in predicting quit success; suitable for frequent monitoring
Frost‐Pineda (2014)	Dependence/addiction	National adult smokers in US cohorts (e.g. Total Exposure Study, *n* > 3500)	Frequency of smoking, cigarettes per day, time to first cigarette, night walking to smoke (plus others)	Dependence, cessation	Moderate	+	Strong: gold standard dependence measures; decades of validation; authoritative sources confirm as ‘gold standard measures’
O'Brien (2022)	Risk perception	Adults (US panel, online survey, *n* = 1642), also validated in younger adults (aged 18–24 years, 39.5%)	Health risk perception (1–5)	Current use, quit intentions, behavioral intentions	Moderate	+	Strong evidence across most psychometric properties except test–re‐test and responsiveness
Mushtaq (2017)	Withdrawal monitoring	Adult smokeless tobacco users (community, *n* = 95)	Heaviness of smokeless tobacco use index and smokeless tobacco dependence index (0–5)	Tobacco Dependence Screener diagnosis, saliva cotinine	Moderate	±	Strong evidence for concurrent validity and criterion validity; reliability limited to internal consistency
Berlin (2013)	Craving/urges	Adults in smoking cessation trial (ADONIS) in France with medical comorbidities (*n* = 310)	Minnesota Nicotine Withdrawal Scale, craving (1–4)	Smoking relapse	Moderate	±	Strong initial support; well developed for practical brief intervention settings
Pienkowski (2022)	Dependence/addiction	Youth and young adults (aged 16–24 years, Canada, n = 1205)	Self‐perceived dependence (very addicted–not at all addicted)	Monthly/daily vaping frequency, nicotine concentration	Low	±	Evidence supports use for cross‐sectional screening and prediction, but lacks longitudinal psychometric evaluation
Bernstein (2016)	Substance use patterns	Adult emergency department patients who smoke (*n* = 666 at 1‐month follow‐up)	7‐day smoking question	Not assessed	Low	±	Strong for concurrent validity; limited other evidence
de Granda‐Orive (2022)	Self‐efficacy/confidence	Smoking cessation clinic patients in Spain/Argentina (*n* = 182)	Khimji–Watts test, item 3; Richmond test, item 4	Abstinence at 1 year	Low	±	Limited: requires further validation; evidence gaps; limitations across psychometric properties

Abbreviations: ADAPT = accelerated development of additive pharmacotherapy treatment; AUD, alcohol use disorder; BUP‐XR = buprenorphine extended release; CID = clinically important difference; DSM = Diagnostic and Statistical Manual of Mental Disorders; GPA = grade point average; ICC = intra‐class correlation coefficient; OUD, opioid use disorder; QoL = quality of life; SUD, substance use disorder; VAS, visual analog scale.

Among studies that reported test–ret‐est reliability, single‐item PROMs showed intra‐class correlation coefficients (ICCs) or kappa coefficients ranging from approximately 0.60 to 0.85, with most values at or above 0.65, indicating at least moderate stability for repeated monitoring (Table 2). Construct validity correlations ranged from small to very large (*r* ≈ 0.11–0.98), with stronger associations observed for craving, treatment readiness, risk perception and self‐efficacy items. Predictive validity estimates were consistently in the expected direction and of clinically meaningful magnitude, with odds ratios typically in the 1.2–3.3 range and as high as 7.3 for treatment readiness and quit intention measures; where reported, discrimination was acceptable (e.g. area under the receiver operating characteristic curve, AUC = 0.71 for early relapse prediction). These coefficients are also reflected in the COSMIN and modified GRADE ratings summarized in Tables 1, 2, 3.

**TABLE 2:** Key performance measures of single‐item patient‐reported outcome measures.

Author (year)	Instrument(s)	Test–re‐test reliability	Construct correlation	Predictive measure of association
Berlin (2013)	MNWS craving	Not reported	Not reported	OR = 1.48 (95% CI = 1.28–1.72) for relapse at week 1; AUC = 0.71 for prediction from post‐cessation craving at week 1
Bertholet (2009)	vas readiness, perception of problems, and taking action	Not reported	SOCRATES factors reported with α = 0.94 and 0.88	VAS highest vs lowest tertile, IRR = 0.57 (95% CI = 0.36–0.91); taking action highest vs lowest quartile, IRR = 0.42 (95% CI = 0.23–0.78); perception of problems 3rd quartile, IRR = 1.94 (1.02–3.68)
Bertholet (2012)	VAS readiness, importance, and confidence	Not reported	Spearman correlations for alcohol scales at baseline: readiness–importance = 0.37; readiness–confidence = 0.21; importance–confidence = 0.13	Alcohol: high vs low importance OR = 2.94 (95% CI = 1.15–7.50) for no unhealthy alcohol use at 6 months; high vs low confidence OR = 2.88 (95% CI = 1.46–5.68). Smoking: among baseline smokers, high vs low confidence OR = 3.29 (95% CI = 1.12–9.62) for no longer smoking at 6 months; readiness and importance not significantly associated with cessation; no significant IRRs for cigarettes/day
Bienkowski (2015)	Readiness VAS	ICC = 0.85	Not reported	Baseline VAS (mean ± SD) = 6.8 ± 3.4 cm. Smokers vs non‐smokers: 3‐month follow‐up, 5.9 ± 3.5 vs 8.7 ± 2.2 cm; 12‐month follow‐up, 6.1 ± 3.6 vs 8.4 ± 2.3 cm (both *P* < 0.01). Cut‐off ≤5 cm: OR = 7.3 (95% CI = 2.0–26.8) for continued smoking at 3 months; OR = 4.9 (95% CI = 1.5–15.9) at 12 months. Sensitivity/specificity for continued smoking at 3 months: 3 cm, 22.8%/93.1% (PPV = 86.7%); 5 cm, 45.6%/89.6% (PPV 89.6%); 9 cm, 75.4%/69.0% (PPV 82.7%)
Blanchard (2023)	Short BAM‐R	Not reported	Not reported	None of the individual items nor the 5‐item subset predicted 90‐day SUD treatment retention or 12‐month mortality; AUC for 90‐day retention = 0.51–0.57; AUC for 12‐month mortality = 0.63–0.66 (age‐driven)
Boyett (2021)	Opioid craving VAS	ICC > 0.70	SOWS item 16, *r* = 0.66–0.79; COWS, *r* = 0.43–0.48 (moderate)	Significant logistic regression (higher opioid craving VAS = increased opioid use; *P* < 0.0001)
Caviness (2013)	Change ladder	Not reported	Multi‐variable linear regression (all predictors standardized): quit desire negatively associated with proportion of days using marijuana (*b* = −0.268, 95% CI = −0.372, −0.166) and with White race (*b* = −0.256, 95% CI = −0.489, −0.037); positively associated with marijuana problem severity (MPS count; *b* = 0.408, 95% CI = 0.302–0.514) and any prior quit attempt (*b* = 0.454, 95% CI = 0.235–0.671)	Cross‐sectional outcome only (motivation score): full model explained 36.9% of variance in quit desire, *F*(13314) = 14.14 (*P* < 0.001)
de Granda‐Orive (2022)	Richmond item 4 Khimji–Watts item 3	Not reported	KWT3 showed significant association with referral source—overall: OV vs AP OR = 2.94 (95% CI = 1.25–7.14); OV vs OE OR = 2.63 (95% CI = 1.15–3.23); men: OV vs AP OR = 5.00 (95% CI = 1.30–20.00); OV vs OE OR = 3.70 (95% CI = 1.03–14.28). RT4 showed no consistent association. No self‐efficacy item predicted abstinence in Cox models	No self‐efficacy item predicted abstinence in Cox proportional hazards models (all RT4 and KWT3 terms NS)
Demmel (2006)	Abstinence, self‐efficacy	Not reported	Correlations among measures (baseline): DTCQ total with abstinence self‐efficacy, *r* = 0.49; with drinking refusal self‐efficacy, *r* = 0.35; with abstinence other‐efficacy, *r* = 0.25; abstinence self‐efficacy with drinking refusal self‐efficacy, *r* = 0.56; with abstinence other‐efficacy, *r* = 0.29; drinking refusal with abstinence other‐efficacy, *r* = 0.21 (all *P* ≤ 0.05)	In multivariate logistic regression (outcome: abstinent vs relapsed at 12 weeks; *n* = 142), only abstinence other‐efficacy predicted outcome: *B* = −0.17 (SE = 0.08), Wald = 4.25, *P* < 0.05, such that lower confidence in others’ success (more pessimistic rating) was associated with abstinence); model *G* ^2^(4) = 10.75, *P* < 0.05, Nagelkerke *R* ^2^ = 0.10
Dollinger (2009)	Alcohol frequency, alcohol quantity, church attendance, prayer, study hours, socializing hours	*r* = 0.84, 0.85, 0.85, 0.86, 0.57, 0.65, respectively	Convergent validity against 14‐day nightly behavioral logs (multi‐method–multi‐trait matrix): single‐item hours of study vs log study days, *r* = 0.41; single‐item hours socializing vs log socializing days, *r* = 0.40; alcohol frequency item vs ‘spent ≥1 hour in bar’, log *r* = 0.53, and vs ‘consumed alcoholic beverage’, log *r* = 0.72; alcohol quantity item vs bar, log *r* = 0.46, and vs beverage, log *r* = 0.56; church attendance vs religious service, log *r* = 0.58; prayer vs private prayer, log *r* = 0.72 (all *P* < 0.001)	Predictive/associative findings: higher GPA correlated with more study (*r* = 0.25), higher father’s education (*r* = 0.20), higher educational goal (*r* = 0.19), higher satisfaction with college (*r* = 0.15), and lower alcohol quantity (*r* = −0.12) and frequency (*r* = −0.15) (all *P* ≤ 0.05)
Eaton (2012)	Drug high VAS	Not reported	Not reported	8.8–10.2 mm corresponded to patient‐perceived ‘a little better’/’a little worse’ categories, defining the CID range for opioid subjective high
Fidler (2010)	Strength of Urges to Smoke (SUTS)	*r* = 0.46	Correlations among dependence measures (*n* = 15 740): SUTS with time‐with‐urges, *r* = 0.68; with FTND, *r* = 0.44; with his, *r* = 0.42; with time‐to‐first‐cigarette, *r* = 0.36; with CPD, *r* = 0.36 (all *P* < 0.001)	SUTS: β = 0.41, Wald = 14.66, OR = 1.51 (95% CI = 1.22–1.86), *P* < 0.001
Frost‐Pineda (2014)	CPD, TTFC, FTND, HSI	Not reported	None of the single‐item FTND indicators (TTFC 4‐category; CPD 4‐category; difficulty not smoking; smoking when ill; ‘cigarette would hate most to give up’; smoking more in first hour) had significant ORs for menthol vs non‐menthol (all 95% CIs crossed 1.0)	Across TES models, menthol smoking did not predict higher FTND scores irrespective of categorization (5 levels, 3 levels, or dichotomized low/medium vs high)
Heather (2008)	Readiness Ruler	Not reported	Concurrent validity vs 12‐item RCQ: Spearman ρ between ruler score (0–4) and RCQ dimensional readiness score = 0.47 (*P* < 0.0005, *n* = 72) overall; association with RCQ stage allocation highly significant (χ^2^(8) = 37.5, *P* < 0.0005; contingency coefficient, *C* = 0.59)	Not reported
Heinz (2006)	Craving VAS	Cocaine VAS mean scores did not change significantly (all *P* NS), heroin VAS showed one small pre–post difference on ‘need heroin’	*r* = 0.38–0.64 (cocaine), *r* = 0.42–0.54 (heroin)	Cocaine: 14‐item and 45‐item CCQ total/factors modestly predicted fewer cocaine‐negative urines (*r* ≈ −0.24, −0.29) and longer retention (Compulsivity factor median 74 vs 47 days, *P* = 0.049); only VAS ‘want cocaine’ weakly predicted use (*r* = −0.28). Heroin: higher HCQ total/factors and VAS ‘crave heroin’ unexpectedly predicted more heroin‐negative urine tests (*r* ≈ 0.21–0.27), and higher lack‐of‐self‐efficacy predicted shorter retention (21 vs 26 days, *P* = 0.038). Multi‐item CCQ/HCQ generally outperformed single‐item VAS; if brevity is required, a 14‐item total score is preferred over a single VAS
Hoeppner (2011)	Self‐efficacy	Not reported	Convergent: single‐item self‐efficacy correlated moderately with ADSES total self‐efficacy (*r* ≈ 0.30–0.56; mean ≈ 0.40) and subscales (*r* ≈ 0.18–0.57, increasing over time), while ADSES subscales inter‐correlated more strongly (*r* ≈ 0.57–0.71). Discriminant: correlations with ADSES temptation scales were similar in magnitude but negative (mean *r* ≈ −0.32), supporting that higher global self‐efficacy relates to lower temptation	For 1‐, 3‐ and 6‐month follow‐ups, higher single‐item self‐efficacy robustly predicted lower relapse risk (β ≈ −0.36, −0.41, *P* < 0.001), increasing model *R* ^2^ (≈0.17–0.23 in basic, 0.32–0.41 with full covariates). When the 20‐item ADSES total or subscales replaced the single item, relapse prediction was weaker and inconsistent, with lower *R* ^2^ (≈0.22–0.28). Authors conclude the 1‐item ruler is more efficient and predictively stronger than the 20‐item scale
Hogue (2010)	Contemplation ladder	Not reported	Convergent: ladder correlated strongly with single‐item motivation rulers (β = 0.57 for desire to quit; β ≈ 0.26 for expected success; β ≈ 0.12 for difficulty and importance; all *P* ≤ 0.05) after covariate adjustment. Concurrent: higher ladder scores related to more prior abstinent days (β = 0.26, *P* < 0.001), being in drug‐free treatment (β = 0.17, *P* < 0.01) and modestly to more prior treatment episodes (β = 0.10, *P* < 0.10)	Over 12 months, each 1‐rung increase on the ladder predicted ≈4% more abstinent days (*B* ≈ 3.9, *P* < 0.001), with significant effects in every quarter (*B* ≈ 3.1–4.4, all *P* ≤ 0.01). It also predicted greater 1‐month treatment engagement (OR for any AOD services ≈ 1.26 per rung) and more days in drug‐free care (trend), with effects present for those in drug‐free or no treatment but not methadone. The ladder remained predictive when controlling for a single‐item ‘desire to quit’, which did not add predictive value
Laudet (2009)	Quality of Life satisfaction	Not reported	Baseline QoL satisfaction correlated with commitment to abstinence and with later QoL change; baseline QoL and motivation intercorrelated (e.g. *r* ≈ 0.20–0.35 across waves)	Each 1‐point higher baseline QoL satisfaction increased odds of sustained remission 1 year later (OR = 1.17, 95% CI = 1.01–1.32) and 2‐year continuous remission (OR = 1.18, 95% CI = 1.03–1.35), controlling for baseline abstinence duration; commitment to abstinence only partially attenuated these effects
Ling (2019)	TEA (substance use, health, lifestyle, community)	ICC = 0.65, 0.71, 0.65, 0.61, respectively	With ASI‐Lite problems, opioid craving VAS, EQ‐5D‐5L and SF‐36 (e.g. TEA health vs EQ‐5D‐5L index, *r* = 0.34; TEA substance vs ASI drug use, *r* ≈ −0.12, −0.38; TEA total vs SF‐36 PCS/MCS, *r* ≈ 0.11–0.25)	TEA total increased from 25.4 ± 9.7 at baseline to 35.0 ± 6.7 at 12 months; change scores differentiated SF‐36 health change groups (improved +13.5 vs no change +11.5 vs declined +8.7). Anchor‐ and distribution‐based MID estimates for TEA total ranged ≈5–8 points
Ludwig (2013)	Self‐efficacy	Not reported	Not reported	OR = 1.5 per point; 28% higher abstinence
Maisto (2011)	Readiness Ruler	Not reported	*r* = 0.16–0.76; *r* = 0.64 with Taking Steps at baseline; *r* = 0.76 at 6 months	Δ*R* ^2^ = 0.07–0.12; β = 0.27, −0.43 (all *P* ≤ 0.01)
Muller (2016)	Quality of life	Not reported	WHOQOL‐BREF overall QoL (*r* = 0.576) and (physical, *r* = 0.452; psychological, *r* = 0.576; social, *r* = 0.499; environment, *r* = 0.452; all *P* < 0.001)	Not reported
Mushtaq (2017)	Time to first dip (TTFD), cans/pouches per week (CPW), dips per day (DPD); used to form brief indices (HSTI, STDI)	Not reported	TTFD associated with withdrawal and higher cotinine levels (tolerance); DPD associated with tolerance and craving; items and derived indices strongly intercorrelated and correlated with longer ST use, higher nicotine concentration and other dependence markers	Not reported
Norberg (2012)	Cones/joints/pipes per day and days/week used	Not reported	Weekly frequency item vs TLFB 90‐day frequency, *r* = 0.73 (*P* < 0.001); quantity item vs TLFB cones/joints/pipes per use day, ICC = 0.92 (95% CI = 0.88–0.94), and vs TLFB grams/day, *r* = 0.90 (*P* < 0.001)	In hierarchical regressions predicting dependence (SDS) and cannabis problems (CPQ), only single‐item quantity and TLFB frequency were significant predictors; TLFB quantity in grams did not add significantly beyond these single‐item measures
O'Brien (2022)	Harm your overall health	Not reported	Overall harm item vs corresponding multi‐item scales: absolute risk, *r* = 0.82; risk vs NRT, *r* = 0.92; risk vs cessation, *r* = 0.98; risk vs cigarettes, *r* = 0.75 (full scale) and *r* = 0.93 (8‐item core)	Higher single‐item risk ratings linked to lower odds of current e‐cig use (OR = 0.39–0.81), higher odds of perceiving e‐cigs as equally/more harmful than cigarettes (OR = 1.11–4.92), higher intentions to quit (B = 0.14–0.60), lower skepticism (B = −0.58, −0.22) and lower intentions to use (B = −0.39, −0.19)
Pienkowski (2022)	Self‐perceived dependence	Not reported	Self‐perceived vs EDS, *r* = 0.719; vs PS‐ECDI, *r* = 0.675; vs time to first vape, *r* = 0.606 (all *P* < 0.001)	Multivariable GLMs (adjusted): predicts vaping frequency, per month (*t* = 7.40, η^2^_p_ = 0.048), per weekend day (*t* = 6.47, η^2^_p_ = 0.037), per weekday (*t* = 6.74, η^2^_p_ = 0.040); and nicotine concentration among nicotine users (*t* = 3.16, η^2^_p_ = 0.010, *P* < 0.01). In head‐to‐head models, the single item was as good as or better than multi‐item indices for several outcomes
Piontek (2017)	VAS readiness	Not reported	Not reported	Multi‐level models: employment → higher ‘can really help’ (*B* = 0.55, *P* < 0.05); education (≥secondary) → lower ‘can really help’ (*B* = −0.74, *P* < 0.05) and lower ‘too demanding’ (*B* = −0.68, *P* = 0.05). Craving (within‐person) → higher ‘too demanding’ (*B* = 0.41, *P* < 0.001) and higher ‘last chance’ (*B* = 0.06, *P* < 0.05). Craving (between‐person) → higher ‘too demanding’ (*B* = 0.28, *P* < 0.001); interactions with time significant for ‘too demanding’
Rosenberg (2007)	VAS (need, desire, craving, urge, compulsion)	Not reported	Inter‐VAS: median *r* = 0.51 (range = 0.35–0.68). With multi‐item craving scales: PACS vs VAS crave, *r* = 0.52; OCDS total vs VAS (crave/urge/compulsion), *r* ≈ 0.32–0.39; AUQ total vs VAS crave/urge *r* ≈ 0.36–0.38; TRI‐CEP vs VAS crave/urge/compulsion, *r* ≈ 0.41–0.50	In simultaneous regressions, VAS ‘urge’ uniquely predicted number of binges in past 2 weeks (standardized β = 0.345, *P* < 0.05); VAS items did not predict drinks per drinking day; models for days/week and binges had *R* ^2^ = 0.537 and 0.438, respectively
Shram (2020)	Drug liking VAS, bad effects VAS, good effects VAS	Not reported	Drug liking Emax strongly correlated with good effects (mean *r* ≈ 0.76) and high (*r* ≈ 0.72); Overall drug liking correlated with take drug again, *r* ≈ 0.82; Drug liking showed little correlation with bad effects; positive‐effect single VAS loaded on a common ‘euphoria/positive effects’ factor (77% variance)	Drug liking Emax/EmaxD effect sizes for active controls typically ≥1.0 and moderately predicted post‐marketing abuse‐related adverse‐event disproportionality (*R* ^2^ ≈ 0.39–0.52, depending on AE term set); take drug again VAS effect sizes were less predictive (*R* ^2^ ≤ 0.30)
Smith (2017)	Readiness to remain abstinent/readiness to stop using	Not reported	Correlations. For quitters: with SES, *r* = 0.229; with TMI, *r* = −0.146; with TRI, *r* = −0.162 (all *P* < 0.05). For non‐quitters: with RFQ, *r* = 0.257 and TMI, *r* = 0.093 (both *P* < 0.05); with TRI, *r* = −0.170 (*P* < 0.05); with SES, *r* = 0.084 (NS)	Logistic regression (adjusted): per 1‐point (1%) increase, AER odds ↑ for quitters at 3 months (OR = 1.01, *P* = 0.003) and 6 months (OR = 1.01, *P* < 0.01); for non‐quitters at 6 months (OR = 1.01, *P* = 0.042). Predicted probability gain from Q3 → Q4 readiness ≈ +19% for quitters (at 3 and 6 months)
Tompkins (2009)	VAS ‘bad effects’, VAS ‘sick’	Not reported	Concurrent validity: peak CINA vs bad effects, *r* = 0.63; vs sick, *r* = 0.65; peak COWS vs each VAS, *r* = 0.57; VAS bad effects vs sick, *r* = 0.88 (all *P* < 0.001)	Not reported
Williams (2007)	Confidence; intention to abstain; readiness, importance, intention to cut down	Not reported	Single‐item means differed by RTCQ stages (all *P* ≤ 0.01). Inter‐item correlations: readiness–importance, *r* = 0.59; readiness–intention to cut down, *r* = 0.53; readiness–intention to abstain, *r* = 0.45; importance–intention to cut down, *r* = 0.69; importance–intention to abstain, *r* = 0.55; confidence showed weak correlation with the others	Multivariable (adjusted) 6‐mo outcomes: Confidence—lower heavy episodic drinking (AOR = 0.88, 95% CI 0.80–0.98), lower risky amounts (AOR = 0.89, 0.79–1.00), and fewer any consequences (AOR = 0.88, 0.79–0.98). Intention to abstain—higher abstinence (AOR = 1.43, 1.09–1.88), lower heavy episodic (AOR = 0.79, 0.64–0.98) and risky amounts (AOR = 0.78, 0.62–0.98). Other single items were not predictive of lower consumption and some associated with more consequences.
Yoon (2021)	Brief Assessment of Cocaine Demand (Q0‐brief, Omax‐brief, Breakpoint‐brief)	Not reported	Q0‐brief–Omax‐brief, *r* = 0.65 (*P* < 0.005); Q0‐brief–Breakpoint, *r* = 0.44 (*P* < 0.05); Omax‐brief–Breakpoint, *r* = 0.75 (*P* < 0.001). Convergent with cocaine‐use indices: Q0‐brief with KMSK lifetime total, *r* = 0.49 (*P* < 0.05) and KMSK lifetime most $, *r* = 0.52 (*P* < 0.05); Omax‐brief with most rocks used in a weekend, *r* = 0.47 (*P* < 0.05), and with most $ spent in a weekend, *r* = 0.71 (*P* < 0.01); Breakpoint‐brief with most $ spent in a weekend, *r* = 0.44 (*P* < 0.05); Q0‐brief with most rocks used in a weekend, *r* = 0.45 (*P* < 0.05)	Not reported

*Note*: Table 2 reports only the statistics provided in each study (ICC/kappa, *r*, OR/IRR/AUC). Given the heterogeneity in models and outcomes, please consult the cited primary studies for full analytic details; ‘Not reported’ indicates that the index was not available. This table is an overview and should be read alongside the COSMIN/modified‐GRADE summaries in the main text.

Abbreviations: ADSES = Alcohol/Drug Self‐Efficacy Scale; AER = abstinent and in early remission; AOD = alcohol and other drugs; aOR = adjusted odds ratio; ASI‐Lite = Addiction Severity Index‐Lite; AUC = area under the curve; AUQ = Alcohol Urge Questionnaire; *B*/β, regression coefficient; BP = Breakpoint; CCQ = Cocaine Craving Questionnaire; CID = clinically important difference; COWS = Clinical Opiate Withdrawal Scale; CPD = cigarettes per day; CPQ = Cannabis Problems Questionnaire; DTCQ = Drug‐Taking Confidence Questionnaire; η^2^_p_, partial eta‐squared; EDS, E‐Cigarette Dependence Scale; Emax = maximum effect; EmaxD = maximum effect at any dose; EQ‐5D‐5L = EuroQol‐5D‐5Level; FTND = Fagerström Test for Nicotine Dependence; GLM = generalized linear model; HCQ = Heroin Craving Questionnaire; HIS = Heaviness of Smoking Index; HSTI = Hooked on Smokeless Tobacco Index; ICC = intra‐class correlation coefficient; IRR = incidence rate ratio; KMSK = Kreek–McHugh–Schluger–Kellogg Scale; KWT3 = Khimji–Watts item 3; MID = minimal important difference; MNWS = Minnesota Nicotine Withdrawal Scale; MPS = marijuana problem severity; NRT = nicotine replacement therapy; OCDS = Obsessive‐Compulsive Drinking Scale; Omax = maximum expenditure; PACS = Penn Alcohol Craving Scale; PPV = positive predictive value; PS‐ECDI = Penn State Electronic Cigarette Dependence Index; Q₀ = demand intensity; QoL, Quality of Life; *R* ^2^ = coefficient of determination; RCQ = Readiness to Change Questionnaire; RFQ = reasons for quitting; RT4 = Richmond test item 4; SES = self‐efficacy scale; SDS = Severity of Dependence Scale; SF‐36 PCS/MCS = Short Form‐36 Physical/Mental Component Summary; SOCRATES = Stages of Change Readiness and Treatment Eagerness Scale; SOWS = Subjective Opiate Withdrawal Scale; STDI = Smokeless Tobacco Dependence Index; TEA = treatment effectiveness assessment; TES = total exposure study; TLFB, timeline follow‐back; TMI = Treatment Motivation Index; TRI = Treatment Resistance Index; TRI‐CEP = Three‐factor Inventory of Craving for Ethanol—Compulsivity/Expectancy/Purpose; VAS, visual analog scale.

**TABLE 3:** Single‐item patient‐reported outcome measures with strongest support.

Author (year)	Question	Response options	Clinical construct	Key outcomes predicted	Key strengths	COSMIN, GRADE
Boyett (2021)	‘With zero (0) meaning “No craving at all” and 100 meaning “strongest craving ever”, please indicate the point on the line that represents your current state’	0–100 mm VAS 0 = ‘no craving at all’ 100 = ‘strongest craving ever’	Craving/urges	Opioid use during treatment	Strong test–re‐test reliability (ICC > 0.70), construct validity with SOWS/COWS, predictive of treatment outcomes	+, High
Bienkowski (2015)	‘How ready are you to quit smoking right now?’	10‐cm vertical VAS ‘not at all’ (bottom)–‘very much’ (top)	Treatment readiness	Smoking cessation at 3–12 months	Excellent test–re‐test reliability (ICC = 0.85), strong predictive validity (OR = 4.9‐7.3)	+, High
Shram (2020)	‘At this moment, my liking for this drug is...’	0–100 point VAS 0 = strong disliking 50 = neither like nor dislike 100 = strong liking	Craving/urges	Post‐market abuse potential	Strong construct validity, known‐groups discrimination, predictive of real‐world abuse rates	+, High
Bertholet (2009)	‘How ready are you to change your drinking habits?’	1–10 VAS 1 = ‘not ready to change’ 10 = ‘completely ready to change’	Treatment readiness	Drinks per day at 3 months	Excellent psychometric properties, factor analysis validation, 42%–58% reduction in drinking	+, Moderate
Demmel (2006)	‘How confident are you that you will not relapse following discharge?’ and ‘How confident are you right now that you could resist the urge to drink?’	0 = ‘not at all confident’ 10 = ‘extremely confident’	Self‐efficacy/confidence	Abstinence/relapse at 12 weeks following discharge	Multi‐variate logistic regression; multiple confidence measures examined for concurrent validity; conservative intention‐to‐treat analysis	+, Moderate
Caviness (2013)	‘On a scale from one to ten, with one representing no desire to quit, give yourself a rating. Choose the number between 1 and 10 that best describes your own desire to stop using marijuana at this time. Remember, the higher the number, the greater your desire	1–10 scale 1 = no desire to quit 10 = greater desire to quit	Treatment readiness	Marijuana use reduction/cessation	Population consisted of women; enrolled emerging adults with highest marijuana use prevalence; racially/ethnically diverse sample	+, Moderate
Norberg (2012)	‘During the past 90 days, approximately how many days a week did you typically smoke cannabis?”’ ‘During the past 90 days, how many cones/joints/pipes did you have on those days when you were using cannabis?”’	Open‐ended (days per week, number of cones, joints, pipes)	Substance use patterns	Cannabis frequency/quantity; dependence	High test–re‐test reliability (*r* = 0.90), strong agreement with timeline follow‐back	+, Moderate
Laudet (2009)	‘In general, how satisfied are you with your life?’ ‘Looking back on the period since the previous interview, would you say that overall, your life has gotten...’	1–10 scale (1 = not at all; 10 = completely) 1–5 scale (1 = gotten much worse; 5 = gotten much better	Quality of life	Sustained remission at 1–2 years	Significant predictor after controlling for remission duration, feasible for repeated use	+, Moderate
Bertholet (2012)	‘How ready are you to change your drinking/smoking habits?”’ ‘How important is it for you right now to change your drinking/smoking?’ ‘If you decide to change your drinking/smoking habits, how confident are you that you would succeed?’	1–10 VAS 1 = ‘not ready’, ‘not important’, ‘not confident’ 10 = ‘ready’, ‘very important’, ‘very confident	Self‐efficacy/confidence	Drinks per day at 3 months	Mandatory recruitment process providing unique opportunity to contact population‐based sample; 80% follow‐up rate in randomized trial	+, Moderate
Heinz (2006)	‘How much are you craving heroin/cocaine?’ ‘How much do you want heroin/cocaine?’ ‘How much do you need heroin/cocaine?’	100‐mm VAS ‘not at all’ (left)–‘extremely’ (right)	Craving/urges	Drug use in treatment	Large sample size; comprehensive assessment using both multi‐dimensional (45‐item) and brief (14‐item) versions; direct comparison of uni‐dimensional vs multi‐dimensional craving measures	+, Moderate
Eaton (2012)	‘How high are you now?’	100‐mm VAS 0 mm ‘not at all’–100 mm ‘extremely”’	Risk perception	Clinical outcome: drug self‐administration; retention	Good validity; both anchor‐based and distribution methods show similar result for clinically important difference; directly applicable to treatment	+, Moderate
Ling (2019)	‘How much better?’ since beginning treatment across the following four domains: substance use; health; lifestyle; community involvement	1–10 for each domain (1 = none/not much; 10 = much better)	Recovery progress	80% abstinent during treatment	All four domains show strong validity and responsiveness	+, Moderate
Fidler (2010)	‘How much of the time have you felt the urge to smoke in the past 24 hours?’ ‘In general, how strong have the urges to smoke been?’	(i) Not at all, (ii) a little of the time, (iii) some of the time, (iv) a lot of the time, (v) almost all of the time, (vi) all of the time; (i) slight, (ii) moderate, (iii) strong, (iv) very strong, (v) extremely strong	Craving/urges	Smoking relapse after quit attempt	Strongest predictor of quit success, outperformed Fagerström Test for Nicotine Dependence and Heaviness of Smoking Index (β = 0.41, *P* < 0.001)	+, Moderate
Frost‐Pineda (2014)	‘How many cigarettes do you smoke per day?’ ‘What is the time between waking and smoking the first cigarette?’	Open‐ended count (0–100+ cigarettes per day) 5 minutes (3 points) 6–30 minutes (2 points) 31–60 minutes (1 point) 60 minutes (0 points)	Dependence/addiction	Nicotine dependence diagnosis	Gold‐standard measures, decades of validation, no menthol/nonmenthol differences	+, Moderate
O'Brien (2022)	‘If you used [product name] every day, how likely is it that you would [health effect]’ ‘If you either used [e‐cig brand] OR cigarettes every day, which product would make it more likely that you would [health effect]’	1 = not at all likely; 4 = very likely 1 = MUCH more likely with e‐cig; 5 = MUCH more likely with cigarettes	Risk perception	E‐cigarette quit intentions; current use	Rigorous content validation with cognitive interviews; high correlation with multi‐item scales	+, Moderate

Abbreviations: ICC = intra‐class correlation coefficient; VAS, visual analog scale.

Of the 35 included studies, 15 achieved an overall rating of sufficient measure properties and moderate or above level‐of‐evidence quality rating across multiple COSMIN domains (Table 3), with ICCs generally of >0.70 and correlations with established scales in the moderate‐to‐large range, as well as evidence of known‐group discrimination and responsiveness to change. The clinical constructs covered among these studies included treatment readiness [62, 63, 64], craving/urges [35, 43, 48, 58], dependence/addiction [57], self‐efficacy/confidence [60, 65], use patterns [53], quality of life [25], risk perception [52, 59] and recovery progress [37]. All measures demonstrated predictive validity for clinically meaningful outcomes, with several yielding odds ratios of >2.0 for key outcomes such as abstinence, relapse or continued smoking, and with some outperforming established multi‐item scales in prediction. Notable examples include a self‐efficacy measure that surpassed a 20‐item scale for general substance relapse [11], a self‐efficacy measure with maximum scores achieving 28% higher prediction for alcohol abstinence than lower scores [55], and a smoking urge measure that outperformed both the Fagerström Test for Nicotine Dependence and the Heaviness of Smoking Index in predicting quit success [58].

**FIGURE 3:**
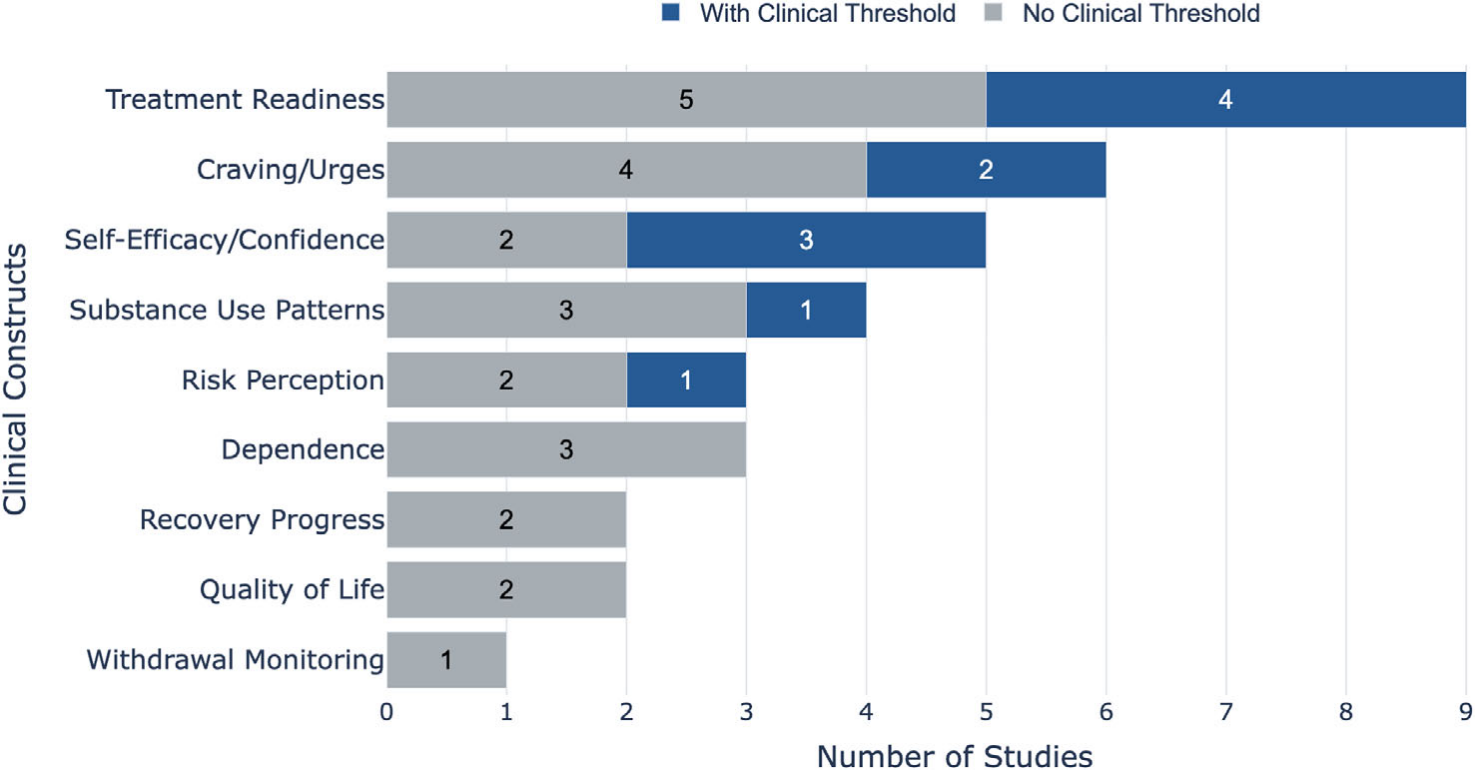
Number of studies by clinical construct. The number in the box indicates the number of studies with/without clinical threshold.

Only 11 studies reported empirically derived thresholds (Figure 3; Table 4) [35, 40, 42, 55, 56, 59, 60, 63, 64, 65, 67]. Among measures with established thresholds, the predominantly predictive cut‐points showed clinical utility, with: the Readiness Ruler demonstrating that scores ≥7 predict higher treatment engagement and better outcomes through empirical modeling [40]; the readiness visual analog scale (VAS) establishing that scores ≤5 indicate a high risk for continued smoking with strong predictive validity [63]; the self‐efficacy measure, identifying 10/10 as the optimal cut‐point for abstinence prediction via receiver operating characteristic analysis [55]; a craving/urges measure of ≥2 on a scale of 1–4 predicting relapse; higher scores on a contemplation ladder scale of 1–7 indicating higher readiness [56]. Anchor‐based methods were used for determining meaningful change, demonstrated by the opioid craving VAS, where a ≥20 mm change represents meaningful improvement based on correlation with established withdrawal scales [35], and also demonstrated by the drug high VAS, where ≥8–10 mm indicates clinically important difference [59]. Distribution‐based and guideline‐derived thresholds were less common, including Bernstein *et al*.’s binary 7‐day smoking question, following established Society for Research on Nicotine and Tobacco (SRNT) task force recommendations [67], and Eaton *et al*.’s drug high VAS, using both anchor and distribution‐based methods to establish ≥8–10 mm as a clinically important difference in the desirability of the drug [59]. However, the majority of measures lacked established clinical thresholds, including several otherwise high‐quality instruments, such as Shram *et al*.’s craving/urges measure [48], Norberg *et al*.’s cannabis use patterns [53] and Laudet *et al*.’s quality of life satisfaction [25].

**TABLE 4:** Clinical cut‐points and interpretation.

Author (year)	Instrument(s)	Clinical construct	Scoring range	Clinical threshold	Method	COSMIN, GRADE
Boyett (2021)	Opioid craving VAS	Craving/urges	0–100 mm	≥20 mm indicates meaningful clinical change	ROC curve, anchor‐based change, predictive modeling	+, High
Maisto (2011)	Readiness Ruler	Treatment readiness	1–10 scale	≥7 predicts higher engagement and better outcomes	Predictive regression, concurrent/predictive validity	±, Moderate
Bertholet (2012)	VAS readiness, importance and confidence	Self‐efficacy/confidence	1–10 scales	≥8 (high category) confidence and importance for alcohol and smoking	Categorical and logistic regression	+, Moderate
Bernstein (2016)	7‐day smoking question	Substance use patterns	Binary (yes/no)	‘No’ = abstinent, ‘Yes’ = non‐abstinent	Kappa agreement, validation vs timeline, biomarker	±, Low
Bienkowski (2015)	Readiness VAS	Treatment readiness	0–10 scale	≤5 indicates high risk of continued smoking	Logistic regression, cut‐off analysis	+, High
Eaton (2012)	Drug high VAS	Risk perception	0–100 mm	≥8–10 mm = clinically important difference	Anchor and distribution‐based	+, Moderate
Ludwig (2013)	Self‐efficacy	Self‐efficacy/confidence	1–10 scale	10/10 cut‐point only for abstinence	ROC curve, dichotomized confidence cut‐point	±, Moderate
Berlin (2013)	Minnesota Nicotine Withdrawal Scale, craving	Craving/urges	1–4 scale	≥2 (mild, moderate and severe categories)	ROC curve analysis	±, Moderate
Bertholet (2009)	VAS readiness, perception of problems and taking action	Treatment readiness	1–10 scales	Tertile/quartile effects shown	Dose–response analysis	+, Moderate
Hogue (2010)	Contemplation ladder	Treatment readiness	1–7 scale	Higher scores predict better outcomes	Predictive validity, regression	±, Moderate
Demmel (2006)	Abstinence self‐efficacy	Self‐efficacy/confidence	0–10 scale categorical grouping: low (1–4); medium (5–7); high (8–10)	≥8–10 (high confidence)	Categorical and logistic regression	+, Moderate

Abbreviations: ROC = receiver operating characteristic; VAS = visual analog scale.

## DISCUSSION

In this systematic review, we identified 35 studies evaluating single‐item PROMs across nine clinical constructs with demonstrated psychometric properties for substance use monitoring. Fifteen studies achieved an overall rating of sufficient measure properties and moderate‐or‐above level of evidence quality rating through COSMIN and modified GRADE evaluation, spanning multiple clinical constructs: treatment readiness, craving/urges, dependence/addiction, self‐efficacy/confidence, use patterns, quality of life, risk perception and recovery progress. Measures assessing craving (e.g. opioid craving VAS), treatment readiness (e.g. Readiness Ruler) and self‐efficacy (e.g. confidence scales) are the most actionable for immediate integration into MBC workflows because of the strong predictive validity, established thresholds and low burden. Several single‐item PROMs outperform multi‐item scales for these constructs, challenging assumptions about the necessity of longer instruments. However, not all substances have validated single‐item PROMs for every construct, reflecting heterogeneity in the evidence base and highlighting gaps for future research. Over half lack empirically validated thresholds and responsiveness, limiting clinical interpretation and monitoring over time. While these tools are practical for routine use, prospective studies to establish meaningful cut‐points and responsiveness to change could be helpful for their adoption in other decision‐making contexts. For routine implementation in MBC, we recommend restricting primary monitoring to single‐item PROMs with at least moderate quality of evidence for reliability, construct validity and predictive validity. Measures with more limited or preliminary evidence may be better suited to research or supplemental monitoring roles until further validation is available.

Our findings extend prior evidence by clarifying when single‐item PROMs meet COSMIN standards for reliability, validity and monitoring utility, and by identifying gaps (e.g. thresholds, responsiveness) that limit implementation. Previous reviews have consistently identified key challenges in the measurement landscape. Santos and colleagues demonstrated that while many multi‐item measures show acceptable psychometric properties, most studies lack diversity in populations and settings, with 84% showing a high risk of bias [28]. Stewart and colleagues revealed that only 18% of identified measures were brief, free and accessible, with just 19% achieving psychometric excellence, predominantly among screening rather than comprehensive assessment tools [16]. Similarly, Migchels and colleagues found substantial variation in PROM implementation approaches, emphasizing the need for standardized, pragmatic solutions that address treatment dropout and staff burden [29]. Bunaciu and colleagues further illustrated this complexity in recovery‐oriented care, identifying 41 distinct constructs across 10 recovery capital measures, while noting limited evidence across diverse populations [30]. Collectively, these reviews underscore the gap between the complexity of available multi‐item instruments and the practical realities of clinical implementation. Our focus on analyzing single‐item PROMs is a step toward addressing the need for pragmatic assessment tools that can overcome barriers to routine implementation while maintaining sufficient validity for clinical decision‐making, particularly in resource‐constrained settings where comprehensive multi‐item assessments may be unfeasible.

Integration with emerging digital health technologies amplifies the potential impact of these findings. Digital MBC systems show high patient engagement rates (with a mean completion of 85.8% of weekly assessments over 24 weeks) and positive usability ratings from both patients and clinicians [68]. Patients report that PROMs enhance self‐reflection, improve patient–clinician communication and extend treatment reach beyond scheduled sessions [69, 70]. Such technology‐enabled approaches address the implementation barriers identified in prior research, including time constraints, staff limitations and competing clinical demands [71]. The integration of single‐item PROMs with electronic health record data may enable real‐time relapse risk assessment and clinical decision support systems [72, 73, 74]. Advanced analytical approaches, including machine learning algorithms, can leverage continuous single‐item data streams to develop personalized risk models and trigger just‐in‐time adaptive interventions [75, 76, 77, 78]. For example, decision tools informed by daily ratings of stress and hopefulness have been shown to predict next‐day alcohol use among emerging adults, enabling interventions to be deployed during high‐risk windows, such as Thursday or Friday evenings, when stress is elevated and hopefulness signals anticipation of drinking [75]. When paired with patient‐facing dashboards or feedback tools, these systems enable individuals to review their own MBC metrics, fostering engagement and shared decision‐making. This convergence of brief assessment, digital delivery and precision medicine approaches represents a paradigm shift toward truly personalized, data‐driven addiction care [79].

### Limitations

The heterogeneity in study designs, populations and reference standards precluded meta‐analytic approaches, limiting the quantitative synthesis of effect sizes. Many studies employed cross‐sectional designs that cannot establish test–re‐test reliability or responsiveness to change over time. The predominant focus on alcohol and tobacco measures reflects historical research priorities rather than current clinical needs, with limited evidence for emerging substances and for the use of multiple substances. Interpretability remains an underdeveloped area, which limits the ability to translate PROM scores into actionable clinical decisions and underscores the critical need for anchor‐based and distribution‐based interpretive guidelines. The absence of established cut‐points and responsiveness to change particularly constrains implementation in MBC systems that require clear decision rules and trajectories for treatment modification. Content validity evidence varied substantially across measures, with many lacking systematic inputs from target populations during development. This limitation is particularly concerning given the emphasis on patient‐centered recovery definitions and the importance of cultural relevance in addiction treatment. Future measure development should prioritize comprehensive stakeholder engagement, including persons with lived experience, to ensure content validity across diverse populations and treatment contexts.

### Future directions

Several critical research priorities emerge from this systematic review. First, longitudinal validation studies are needed to establish test–re‐test reliability and responsiveness to change for promising single‐item PROMs [16]. Such studies should employ multiple timepoints across treatment trajectories to capture both short‐term fluctuations and longer‐term recovery patterns [80]. Responsiveness research should incorporate both anchor‐based and distribution‐based approaches to establish minimally important differences that support clinical decision‐making [81, 82]. Second, comprehensive psychometric evaluation using modern measurement approaches is warranted. Item response theory analyses can optimize response scale formats and establish precise measurement across the full continuum of severity [83]. Differential item functioning studies should examine measurement invariance across key demographic groups, substance types and treatment settings to ensure equitable application [84]. Such analyses are particularly important given the diverse populations served in addiction treatment. Third, implementation science research should investigate optimal integration strategies for single‐item PROMs within existing clinical workflows. Mixed‐methods studies examining barriers and facilitators to adoption can inform tailored implementation strategies that address organizational, provider‐ and patient‐level factors [85, 86, 87]. Economic evaluation of MBC using single‐item PROMs should quantify cost‐effectiveness compared with traditional approaches and longer assessment batteries [88, 89]. Fourth, technology‐enhanced assessment approaches warrant systematic investigation. Digital delivery platforms offer opportunities for real‐time data collection, automated scoring and integration with clinical decision support systems [90, 91, 92]. Research should examine optimal delivery modalities, frequency of assessment and integration with ecological momentary assessment approaches that capture dynamic recovery processes [93]. Fifth, precision medicine applications of single‐item PROMs represent a promising area of research [27]. Machine learning approaches can identify patterns in continuous single‐item data streams to predict relapse risk, optimize treatment intensity and personalize intervention timing [94].

### Policy and practice implications

Single‐item PROMs offer practical pathways for implementing evidence‐based quality measurement in addiction treatment. Quality initiatives should prioritize outcome‐based measures reflecting meaningful recovery constructs over process indicators with limited clinical benefit. These brief measures provide psychometrically sound yet operationally feasible options for routine monitoring. For implementation, a pragmatic approach is to deploy a brief core set combining: (i) craving/urge intensity [35]; (ii) readiness or confidence/self‐efficacy [11]; and (iii) a patient‐centered functioning/recovery indicator (e.g. recovery progress or quality‐of‐life satisfaction) [25, 37], tailored to the clinical setting (Box 2). This combination supports near‐term risk detection, treatment planning and recovery‐oriented monitoring while maintaining feasibility for routine MBC.

Box 2 **Practical guidance for single‐item patient‐reported outcome measures (PROMs) in measurement‐based care (MBC).**

**Use as core, primary monitoring (strongest evidence)**

- Craving/urge items (e.g. opioid or tobacco craving visual analog scale, VAS) for frequent monitoring and near‐term risk detection [35]
- Readiness/motivation rulers or VAS for guiding engagement and treatment planning [63, 64]
- Confidence/self‐efficacy items for relapse prevention planning and shared decision‐making [11, 65]

**Use as supplemental monitoring (promising but less mature evidence)**

- Quality of life and recovery progress items as patient‐centered outcomes alongside core monitoring [25, 37]
- Risk perception and dependence/addiction items to enrich assessment, interpreted cautiously across settings [51, 52]
- Withdrawal items when clinically indicated rather than as universal core measures [47, 54]

**When thresholds are not available**

- Rely on within‐person trends from baseline and pair changes with clinical anchors (e.g. lapses, missed visits, medication changes) to guide follow‐up and stepped care.

As many single‐item PROMs lack empirically validated cut‐points, implementation can rely on within‐person trending (change from an individualized baseline) and pre‐specified escalation rules (e.g. sustained worsening across consecutive assessments triggers clinical follow‐up). Until standardized thresholds are established, pairing PROM trends with clinical anchors (e.g. recent use episodes, missed visits, medication changes or patient goals) can support actionable MBC. Policy reforms could expand accreditation standards (e.g. Joint Commission and URAC) to include MBC across treatment settings, with specific psychometric criteria and implementation guidance. Value‐based payment models incorporating patient‐reported outcomes could align financial incentives with recovery‐oriented care, particularly given the low administrative burden of single‐item PROMs. Practice transformation would require workforce training emphasizing the clinical utility of outcome data for treatment planning, rather than viewing assessment as an administrative burden. Training should also prepare clinicians to select, administer and interpret brief PROMs effectively, supporting the systematic outcome monitoring that enhances both treatment quality and patient‐centered care. As there is limited evidence on content validity across diverse groups, implementation should include attention to cultural/linguistic appropriateness and local validation, particularly in under‐resourced settings where feasibility disadvantages are most relevant.

## CONCLUSION

Single‐item PROMs are a practical solution to implementing MBC in substance use treatment. This systematic review demonstrates that carefully developed single‐item PROMs can achieve psychometric performance comparable with multi‐item scales, while reducing assessment burden. The convergence of brief assessment tools, digital health technologies, and precision medicine approaches offers opportunities to enhance treatment quality and outcomes through routine patient‐reported outcome monitoring. Importantly, these technologies create the potential for patients to actively review and track their own data over time, fostering engagement, self‐awareness and collaborative decision‐making. However, realizing this potential requires continued research to address interpretability gaps, establish implementation best practices and develop the technological infrastructure necessary to support widespread adoption. The overarching goal is a treatment system where MBC becomes routine and essential in medical practice, supporting clinicians and patients in delivering personalized, evidence‐based care that optimizes recovery outcomes for all individuals seeking treatment for substance use.

## AUTHOR CONTRIBUTIONS

**Thomas J. Reese:** Conceptualization; methodology; data curation; investigation; formal analysis; funding acquisition; visualization; resources; writing—original draft; writing—review and editing. **Hilary A. Tindle:** Investigation; supervision; writing—review and editing. **Justin Bachmann:** Investigation; writing—review and editing. **Adam Wright:** Investigation; writing—review and editing. **Jessica S. Ancker:** Investigation; writing—review and editing. **Carolyn M. Audet:** Investigation; writing—review and editing. **Mauli V. Shah:** Data curation; validation; writing—review and editing; project administration; investigation. **Bryan D. Steitz:** Investigation; writing—review and editing. **Michael H. Levin:** Investigation; writing—review and editing. **Kristopher A. Kast:** Investigation; writing—review and editing. **David Marcovitz:** Investigation; writing—review and editing. **Amanda von Horn:** Investigation; writing—review and editing. **A. Taylor Kelley:** Investigation; writing—review and editing. **John F. P. Bridges:** Conceptualization; methodology; visualization; supervision; investigation; writing—review and editing.

## ACKNOWLEDGMENTS

This study is funded by the Agency for Healthcare Research and Quality (K08HS029695).

## DECLARATION OF INTERESTS

The authors declare no relevant conflicts of interest or financial relationships.

## Supporting information


**Figure S1.** PRISMA flow diagram for included studies.

**Table S1.** PubMed search strategy for MEDLINE.

## Data Availability

The data that support the findings of this study are available from the corresponding author upon reasonable request.
