# Supplementary material for: Patient‐reported outcomes for monitoring substance use treatment: A systematic review of single‐item measures
Source: Addiction. 2026 Apr 22;121(9):2292–316. doi: 10.1111/add.70424 (PMC13486943; doi:10.1111/add.70424)
Supplement: Supplementary file 1 [file ADD-121-2292-s001.pdf]

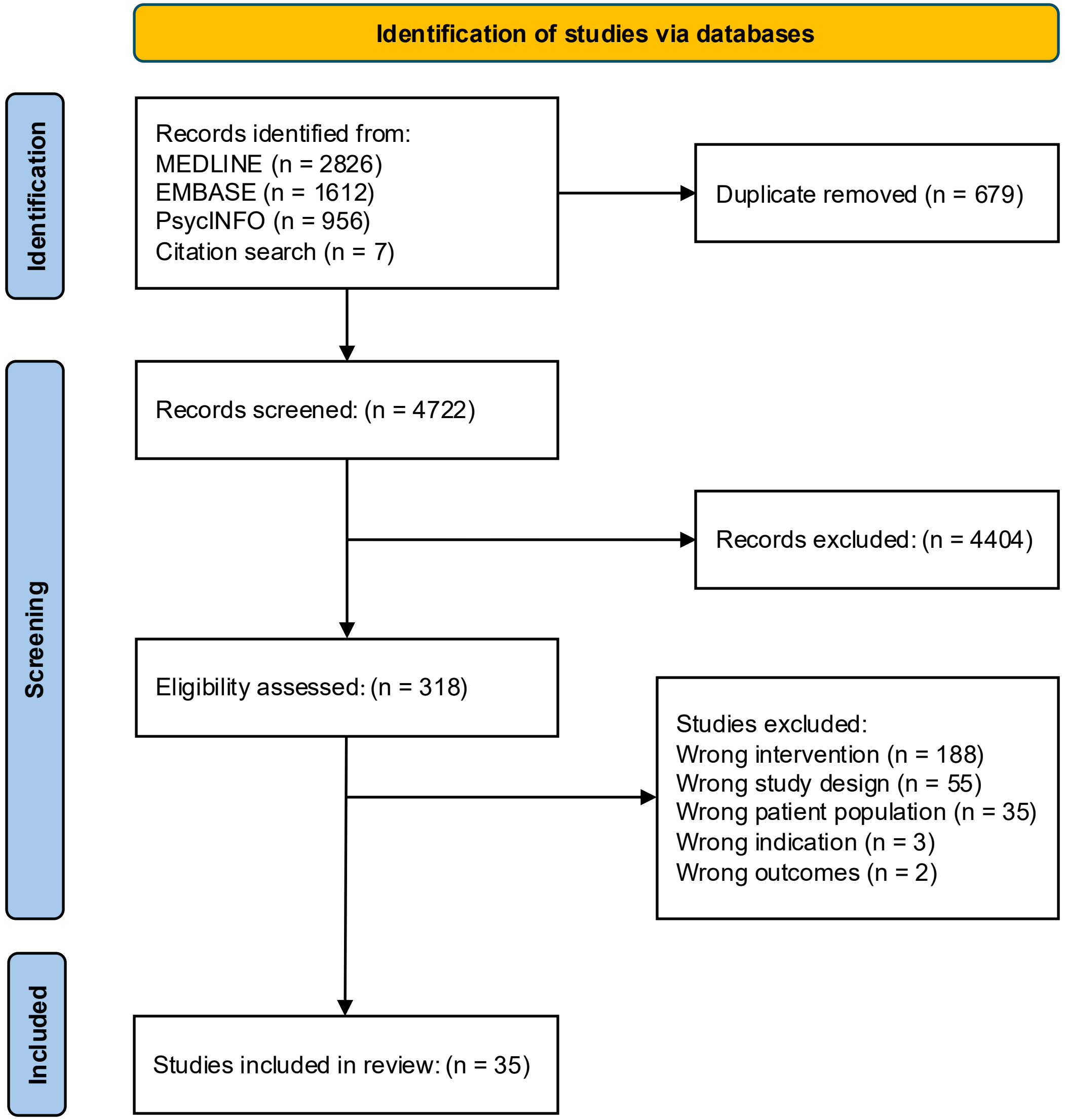
Identification of studies via databases
Identification
Records identified from:
MEDLINE (n = 2826)
EMBASE (n = 1612)
PsycINFO (n = 956)
Citation search (n = 7)
Duplicate removed (n = 679)
Screening
Records screened: (n = 4722)
Records excluded: (n = 4404)
Eligibility assessed: (n = 318)
Studies excluded:
Wrong intervention (n = 188)
Wrong study design (n = 55)
Wrong patient population (n = 35)
Wrong indication (n = 3)
Wrong outcomes (n = 2)
Included
Studies included in review: (n = 35)
