# Supplementary material for: Patient‐reported outcomes for monitoring substance use treatment: A systematic review of single‐item measures
Source: Addiction. 2026 Apr 22;121(9):2292–316. doi: 10.1111/add.70424 (PMC13486943; doi:10.1111/add.70424)
Supplement: Supplementary file 2 [file ADD-121-2292-s002.docx]

| Supplement Table 1. PubMed Search Strategy for MEDLINE | |
| --- | --- |
| Construct | ("single item*"[tiab] OR "single-item*"[tiab] OR "one item*"[tiab] OR "brief measure*"[tiab] OR "brief assessment*"[tiab] OR "brief scale*"[tiab] OR "short measure*"[tiab] OR "visual analog* scale*"[tiab] OR "visual analogue scale*"[tiab] OR VAS[tiab] OR "rating scale*"[tiab] OR "readiness ruler*"[tiab] OR "single question*"[tiab] OR "one question*"[tiab] OR "ultra-brief*"[tiab] OR "minimal assessment*"[tiab] OR "rapid assessment*"[tiab] OR "quick measure*"[tiab]) |
|  | **AND** |
| Population | ("Substance-Related Disorders"[MeSH] OR "Substance-Related Disorders/therapy"[MeSH] OR "Alcohol-Related Disorders"[MeSH] OR "Alcohol Drinking/psychology"[Mesh] OR "Opioid-Related Disorders"[MeSH] OR "Cocaine-Related Disorders"[MeSH] OR "Marijuana Abuse"[MeSH] OR "Tobacco Use Disorder"[MeSH] OR "substance use disorder*"[tiab] OR "substance abuse"[tiab] OR "substance dependence"[tiab] OR "drug dependence"[tiab] OR "drug addiction"[tiab] OR alcoholism[tiab] OR "alcohol use disorder"[tiab] OR "alcohol dependence"[tiab] OR "alcohol abuse"[tiab] OR "opioid use disorder"[tiab] OR "opioid dependence"[tiab] OR "cannabis use disorder"[tiab] OR "cocaine dependence"[tiab] OR "tobacco use disorder"[tiab] OR "nicotine dependence"[tiab] OR "illicit drug*"[tiab] OR "hazardous drinking"[tiab] OR "unhealthy alcohol use"[tiab] OR "problem drinking"[tiab] OR smoking[tiab] OR smoker*[tiab]) |
|  | **AND** |
| Instrument | ("Patient Reported Outcome Measures"[MeSH] OR "Surveys and Questionnaires"[MeSH] OR "Self Report"[MeSH] OR "Health Status Indicators"[MeSH] OR "Treatment Outcome"[MeSH] OR "Patient Care Management"[MeSH] OR "Disease Management"[MeSH] OR "Visual Analog Scale"[MeSH] OR "Quality of Life"[MeSH] OR "treatment monitoring"[tiab] OR "clinical monitoring"[tiab] OR "outcome monitoring"[tiab] OR "progress monitoring"[tiab] OR "treatment outcome*"[tiab] OR "treatment evaluation"[tiab] OR "measurement based care"[tiab] OR "measurement-based care"[tiab] OR MBC[tiab] OR "outcome assessment"[tiab] OR "clinical assessment"[tiab] OR "therapy evaluation"[tiab] OR "follow up"[tiab] OR "follow-up"[tiab] OR longitudinal[tiab] OR questionnaire*[tiab] OR "rating scale*"[tiab] OR "assessment tool*"[tiab] OR "evaluation instrument*"[tiab] OR "patient-reported outcomes"[tiab]) |
|  | **AND** |
| Measurement properties filter | (instrumentation[sh] OR methods[sh] OR "Validation Study" [Publication Type] OR "Comparative Study"[pt] OR "psychometrics"[MeSH] OR psychometr*[tiab] OR clinimetr*[tw] OR clinometr*[tw] OR "Outcome Assessment, Health Care"[Mesh] OR "outcome assessment"[tiab] OR "outcome measure*"[tw] OR "observer variation"[MeSH] OR "observer variation"[tiab] OR "Health Status Indicators"[Mesh] OR "reproducibility of results"[MeSH] OR reproducib*[tiab] OR "discriminant analysis"[MeSH] OR reliab*[tiab] OR unreliab*[tiab] OR valid*[tiab] OR "coefficient of variation"[tiab] OR coefficient[tiab] OR homogeneity[tiab] OR homogeneous[tiab] OR "internal consistency"[tiab] OR (cronbach*[tiab] AND (alpha[tiab] OR alphas[tiab])) OR (item[tiab] AND (correlation*[tiab] OR selection*[tiab] OR reduction*[tiab])) OR agreement[tw] OR precision[tw] OR imprecision[tw] OR "precise values"[tw] OR test-retest[tiab] OR (test[tiab] AND retest[tiab]) OR (reliab*[tiab] AND (test[tiab] OR retest[tiab])) OR stability[tiab] OR interrater[tiab] OR inter-rater[tiab] OR intrarater[tiab] OR intra-rater[tiab] OR intertester[tiab] OR inter-tester[tiab] OR intratester[tiab] OR intra-tester[tiab] OR interobserver[tiab] OR inter-observer[tiab] OR intraobserver[tiab] OR intra-observer[tiab] OR intertechnician[tiab] OR inter-technician[tiab] OR intratechnician[tiab] OR intra-technician[tiab] OR interexaminer[tiab] OR inter-examiner[tiab] OR intraexaminer[tiab] OR intra-examiner[tiab] OR interassay[tiab] OR inter-assay[tiab] OR intraassay[tiab] OR intra-assay[tiab] OR interindividual[tiab] OR inter-individual[tiab] OR intraindividual[tiab] OR intra-individual[tiab] OR interparticipant[tiab] OR inter-participant[tiab] OR intraparticipant[tiab] OR intra-participant[tiab] OR kappa[tiab] OR kappa's[tiab] OR kappas[tiab] OR repeatab*[tw] OR ((replicab*[tw] OR repeated[tw]) AND (measure[tw] OR measures[tw] OR findings[tw] OR result[tw] OR results[tw] OR test[tw] OR tests[tw])) OR generaliza*[tiab] OR generalisa*[tiab] OR concordance[tiab] OR (intraclass[tiab] AND correlation*[tiab]) OR discriminative[tiab] OR "known group"[tiab] OR "factor analysis"[tiab] OR "factor analyses"[tiab] OR "factor structure"[tiab] OR "factor structures"[tiab] OR dimension*[tiab] OR subscale*[tiab] OR (multitrait[tiab] AND scaling[tiab] AND (analysis[tiab] OR analyses[tiab])) OR "item discriminant"[tiab] OR "interscale correlation*"[tiab] OR error[tiab] OR errors[tiab] OR "individual variability"[tiab] OR "interval variability"[tiab] OR "rate variability"[tiab] OR (variability[tiab] AND (analysis[tiab] OR values[tiab])) OR (uncertainty[tiab] AND (measurement[tiab] OR measuring[tiab])) OR "standard error of measurement"[tiab] OR sensitiv*[tiab] OR responsive*[tiab] OR (limit[tiab] AND detection[tiab]) OR "minimal detectable concentration"[tiab] OR interpretab*[tiab] OR ((minimal[tiab] OR minimally[tiab] OR clinical[tiab] OR clinically[tiab]) AND (important[tiab] OR significant[tiab] OR detectable[tiab]) AND (change[tiab] OR difference[tiab])) OR (small*[tiab] AND (real[tiab] OR detectable[tiab]) AND (change[tiab] OR difference[tiab])) OR "meaningful change"[tiab] OR "ceiling effect"[tiab] OR "floor effect"[tiab] OR "Item response model"[tiab] OR IRT[tiab] OR Rasch[tiab] OR "Differential item functioning"[tiab] OR DIF[tiab] OR "computer adaptive testing"[tiab] OR "item bank"[tiab] OR "cross-cultural equivalence"[tiab]) |
|  | **NOT** |
| Exclusion filters | ("address"[Publication Type] OR "biography"[Publication Type] OR "case reports"[Publication Type] OR "comment"[Publication Type] OR "directory"[Publication Type] OR "editorial"[Publication Type] OR "festschrift"[Publication Type] OR "interview"[Publication Type] OR "lecture"[Publication Type] OR "legal case"[Publication Type] OR "legislation"[Publication Type] OR "letter"[Publication Type] OR "news"[Publication Type] OR "newspaper article"[Publication Type] OR "patient education handout"[Publication Type] OR "popular work"[Publication Type] OR "congress"[Publication Type] OR "consensus development conference"[Publication Type] OR "consensus development conference, nih"[Publication Type] OR "practice guideline"[Publication Type]) NOT ("animals"[MeSH Terms] NOT "humans"[MeSH Terms]) NOT("screening"[tiab] OR "screen"[tiab] OR "case finding"[tiab] OR "diagnostic test*"[tiab] OR "diagnostic assessment"[tiab]) |
|  | **AND** |
|  | ("2005/01/01"[PDAT] : "2025/08/01"[PDAT]) |
|  | **AND** |
|  | ("adult"[MeSH] OR adult*[tiab]) |
|  | **NOT** |
|  | ("child"[MeSH] OR child*[tiab] OR pediatric*[tiab]) |
